# Streptococcal toxic shock syndrome in a patient with community-acquired pneumonia. Impact of rapid diagnostics on patient management

**DOI:** 10.1099/acmi.0.000144

**Published:** 2020-06-17

**Authors:** Kristian Bagge, Louise Pedersen, Jan Gorm Lisby

**Affiliations:** ^1^​ Department of Clinical Microbiology, Hvidovre Hospital, University of Copenhagen, Hvidovre, Denmark; ^2^​ Department of Anesthesiology and Intensive Care, Bispebjerg Hospital, University of Copenhagen, Copenhagen, Denmark

**Keywords:** biofire film array, community-acquired pneumonia, point-of-care, streptococcal toxic shock syndrome, *Streptococcus pyogenes*, syndromic testing

## Abstract

Here we describe a community-acquired pneumonia with *Streptococcus pyogenes *, group A following a common cold caused by human metapneumovirus. The patient, a 58-year-old woman with no prior medical history, developed respiratory failure and multi-organ dysfunction caused by streptococcal toxic shock syndrome. The patient was admitted to the intensive care unit and treated with supportive care. The definitive diagnosis was made by BioFire FilmArray by Biomerieux (multiplex PCR) 12 h before positive blood culture, thus enabling the clinician to add clindamycin and intravenous immunoglobulin to the treatment. The patient was discharged fully recovered after 23 days. Streptococci group A is a rare pathogen of severe pneumonia and rapid diagnostics by syndromic testing, potentially performed in a near patient setting, is crucial for early implementation of targeted antimicrobial treatment.

## Introduction

This case describes a rare cause of severe community-acquired pneumonia. The patient had streptococcal toxic shock syndrome, a clinical condition with rapid onset and high mortality. As targeted treatment for streptococcal toxic shock syndrome caused by *Streptococcus pyogenes* differs from the empirical treatment, and as targeted treatment for this clinical syndrome is possible based upon microbial identification alone, rapid microbial diagnostics is important. In the described case, a new commercially available BioFire FilmArray Pneumonia Panel enabled definitive microbial diagnosis 12 h before blood cultures were reported as positive.

## Case report

A 58-year-old woman with no prior medical history was admitted to the intensive care unit (ICU) due to septic shock and multi-organ dysfunction syndrome (MODS) caused by streptococcal toxic shock syndrome (STSS).

The patient had suffered from a cough and common cold for 1 week progressing into dyspnea and respiratory failure prior to admission. Due to hypoxia despite supplemental oxygen therapy in the emergency department the patient was transferred to the ICU where she was intubated and put on ventilator support.

Over the course of a few hours, the patient developed profound septic shock with MODS including septic cardiomyopathy and vasoplegia with severe hypotension requiring high doses of norepinephrine, epinephrine and dopamine, acute kidney injury (AKI), progressive respiratory failure and coagulopathy. Continuous renal replacement therapy and shock reversal therapy with high-dose corticosteroid was initiated a few hours after arrival.

### Investigations

When transferred to the ICU the patient was leucopenic with a total white blood cell (WBC) count of less than 0.5×10^9^ l^−1^, C-Reactive Protein (CRP) 218 mg l^−1^ and procalcitonin (PCT) 60.5 µg l^−1^.

Arterial blood gasses showed metabolic lactate acidosis with pH 7.12, lactate 3.4 mmol l^−1^ and base excess (BE) −12.7 mmol l^−1^.

**Fig. 1.**
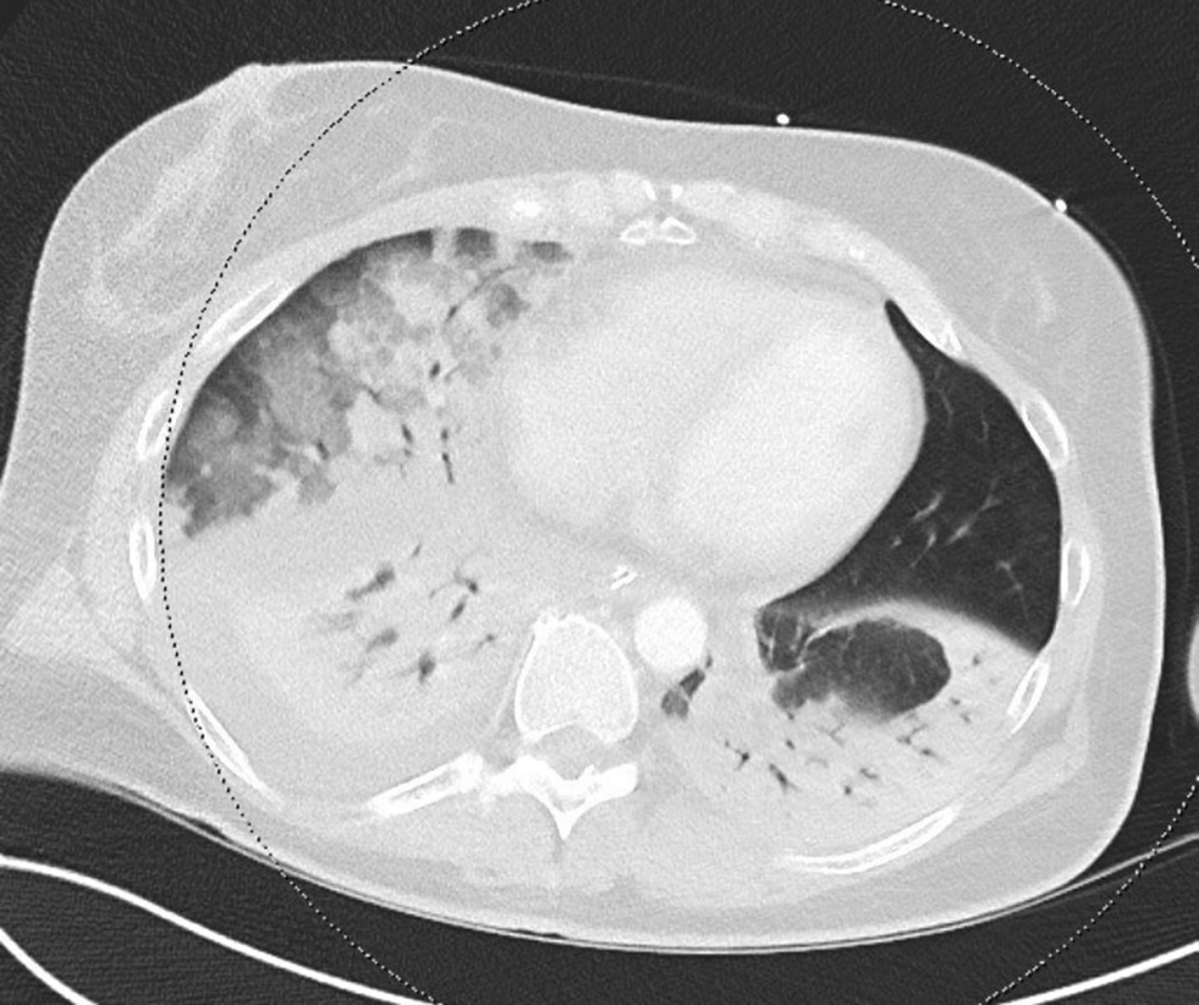
Thoracic CT scan showing bilateral pneumonic infiltrations and right-side pleural effusion.

A thoracic CT scan (Fig. 1) showed bilateral lobular pneumonia and minor pleural effusion.

Tracheal secretion aspirates sent for microbiological cultures using BioFire FilmArray Pneumonia Panel (BioFire) was positive for haemolytic *Streptococcus pyogenes *, group A (GAS) and human metapneumovirus (HMPV) enabling the clinician to target antibiotic treatment within hours of sampling. GAS was subsequently found in blood culture.

### Diagnosis

The severity of the patients’ symptoms escalated quickly after hospital admission with respiratory failure progressing to MODS. Prior to that, the patient had a catarrhal stage. At admission the patient was in septic shock, and thus bacteremia was expected, with pulmonary focus considered most likely. The respiratory failure could be secondary to the septic state, and another focus such as pyelonephritis or a focus secondary to viral infection like otitis media or sinusitis was possible. The respiratory deterioration could have been due to a concurrent pulmonary embolism, which was ruled out by a CT scan. Community-acquired pneumonia in a healthy individual is most frequent due to *Streptococcus pneumonia*. With a prior virus infection and rapid onset of severity *Staphylococcus aureus* superinfection also needs to be taken into consideration when choosing empiric antibiotic treatment. As the patient was an American tourist, the infection could have been acquired before arrival in Denmark. The patients’ travel history and the prevalence of antibiotic-resistant strains should be kept in mind. Our patient had GAS pneumonia, which is seen less frequently, but the condition is well described in the literature as a post-viral complication.

### Treatment

The patient was treated in the emergency room with intravenous benzylpenicillin 1.2 g QID and clarithromycin 500 mg BID, according to local guidelines. As she clinically deteriorated, penicillin was substituted with meropenem 2 g TID and clarithromycin with ciprofloxacin 400 mg TID, primarily to cover for *S. aureus* and penicillin-resistant pneumococci, which we in Denmark only see as imported cases. Retrospectively, the patient could also have had a MRSA pneumonia, in which case meropenem and ciprofloxacin would not have been the optimal treatment. At the time of microbial GAS diagnosis, intravenous clindamycin 600 mg TID was added and a daily dose of 20 g intravenous immunoglobulin (IVGI) was supplemented for a total of 3 days. Once the patient was out of septic shock, the final treatment was deescalated to benzylpenicillin 3 g QID.

### Outcome and follow up

Over the following days, the patient stabilized and inopressors were discontinued after 72 h. Extubation was performed after 5 days preceded by pleural drainage by a pigtail catheter in the right pleural cavity. Kidney function was regained, and continuous renal replacement therapy discontinued after 6 days.

The patient was finally discharged to outpatient follow up after a total of 23 days of hospitalization. At the time of discharge, the patient was fully recovered, albeit still suffering from general fatigue due to the prolonged immobilization.

## Discussion

*Streptococcus pyogenes* is Gram-positive β-haemolytic streptococcus associated with diverse infections in humans, most common pharyngitis and skin and soft tissue infections. In the mid-twentieth century it used to be a common cause of community-acquired pneumonia, often seen in outbreaks, but is now considered a rare aetiology [1]. Invasive GAS infections can be accompanied by STSS, which was defined in 1993 by a working group after various cases had been described in the 1980s of infections with high-virulence GAS strains [2–4]. STSS is defined by a positive culture of GAS, hypotension and end-organ failure [3]. It has a rapid onset with severe morbidity and high mortality, in some studies up to 25 % within the first 24 h [1, 5]. Although GAS is sensitive to penicillin, in the case of STSS, studies have shown a substantially better outcome when clindamycin and IVGI is added to the treatment with a β-lactam antibiotic. A European randomized controlled study has shown an effect of IVGI on STSS, with a 3.6-fold higher mortality rate in the placebo group [6]. The findings were significant, but the study had to be terminated preliminary due to slow enrollment. A meta-analysis has recently come to the same conclusion, including the randomized study and four non-randomized studies [5]. The studies were primarily focused on soft-tissue infections, and only few STSS pneumonias were included. Partly due to the low incidence, there are not any studies showing if IVGI also leads to better patient outcome specifically in STSS pneumonia. In recent published case stories, it is not consistent if the patient received IVGI or not [7]. We believe based on the present evidence and the high mortality that IVGI should also be used in the treatment of STSS pneumonia. Substantial lower mortality rates have also been found when adding clindamycin to STSS treatment [8, 9]. Clindamycin has better tissue penetration than penicillin and inhibits the 50S bacterial ribosome thus inhibiting toxin production. Our patient was also diagnosed with HMPV. This is in concordance with other reports, that GAS pneumonia is often seen post-viral [1, 7].

Due to the severity and rapid onset of the infection, and because the definitive treatment differs from the empirical, rapid diagnostics are crucial. In our case, testing patient material by BioFire FilmArray Pneumonia Panel made definitive diagnosis possible 12 h before blood cultures were reported as positive and thereby made it possible to add clindamycin and IVGI earlier than conventional culture could support, which given the high mortality, especially within the first 24 h, potentially could have affected the patient outcome. Multiplex (syndromic) PCR is a promising tool for rapid analysis of tracheal sputum in patients presenting with severe pneumonia [10–13]. Different test panels exist on the market, each made with specific combinations of viruses, bacteria and genotypic markers of resistance [10]. In general it is accepted that the method detects more pathogens than conventional culture [10–13], but no studies have so far translated this into improved clinical outcome or better usage of antibiotics [10]. The higher sensitivity may result in difficulties interpreting the result, one study found more samples positive for *Staphylococcus aureus* and *Haemophilus influenzae* compared to culture, where the found pathogen might have been a colonizer of the respiratory tract [12]. Another drawback is cost, as the test is relatively expensive. Moreover, since pathogens not included in the panel will not be found, the test may only be used as a supplement to conventional culture. Finally, although not the case here, as all *Streptococcus pyogenes* isolates are penicillin susceptible, for most detected pathogens, a conventional phenotypic antimicrobial susceptibility test will be necessary to determine the optimal targeted antimicrobial treatment. Implementation in the clinical setting is still in its early stages and even though the technique has the potential to facilitate antibiotic stewardship and identify patients that are not covered by the empirical treatment, optimal usage of the test has not yet been defined. Future implementation could potentially be in a near-patient setting (point of care) to further shorten time to microbial diagnosis. A syndromic test for lower respiratory tract infections should in our opinion include group A streptococci.
